# Investigation of outbreak cases infected with the SARS-CoV-2 B.1.640 variant in a fully vaccinated elderly population, Normandy, France, November to December 2021

**DOI:** 10.2807/1560-7917.ES.2022.27.6.2200078

**Published:** 2022-02-10

**Authors:** Brice Mastrovito, Chloé Naimi, Leslie Kouam, Xavier Naudot, Lucie Fournier, Guillaume Spaccaferri, Jean-Christophe Plantier, Anaïs Soares, Fabienne De Oliveira, Marie Gueudin, Véronique Jacomo, Céline Leroy, Alice Moisan, Mélanie Martel

**Affiliations:** 1 Regional Unit Normandy, Santé Publique France, Rouen, France; 2 Infectious Diseases Department, Eurofins-Biomnis, Lyon, France; 3 Department of Infectious Diseases, Santé publique France, Saint Maurice, France; 4 Santé publique France, Saint Maurice, France; 5 Normandie Univ, UNIROUEN, UNICAEN, INSERM DYNAMICURE, CHU Rouen, Department of Virology, Rouen, France; 6 Agence Régionale de Santé Normandie, Caen, France

**Keywords:** SARS-CoV-2, COVID-19, Cluster, Outbreak investigation, Epidemiology

## Abstract

Three confirmed infections with the SARS-CoV-2 B.1.640 variant under monitoring were reported in Normandy, north-western France in late November 2021. Investigations led to the identification of two events linked to the same cluster. A total of 75 confirmed and probable B.1.640 cases were reported. All had completed the primary vaccination series. Sixty-two cases were older than 65 years. Fifty-six cases had symptoms and four were hospitalised. This investigation provides preliminary results concerning a variant with limited information currently available.

On 30 November 2021, the Rouen University Hospital informed the Agence Régionale de Santé (ARS, regional Ministry of Health offices) and Santé publique France (SpF, the French national public health agency) of the sequencing of three cases of a severe acute respiratory syndrome coronavirus 2 (SARS-CoV-2) variant under monitoring: Phylogenetic Assignment of Named Global Outbreak (Pango) lineage B.1.640 [1,2]. One of them had attended a community event for elderly people 2 weeks earlier. Santé publique France and ARS started the investigation immediately upon notification.

## Epidemiological investigations

In this investigation, a confirmed B.1.640 case was a person in whom the SARS-CoV-2 B.1.640 variant had been identified by whole genome sequencing. A probable B.1.640 case was any person with SARS-CoV-2 infection confirmed by RT-qPCR or antigen test and with ascertained at-risk contact with a confirmed case (B.1.640) within 14 days before symptom onset or before a positive test. A non-case was any person with a negative SARS-CoV-2 test confirmed by RT-qPCR or antigen test or a person that was not tested.

Investigations were conducted by SpF with the assistance of the ARS. Interviews with two of the first three cases with sequenced SARS-CoV-2 B.1.640 identified two events linked to the same cluster. Participants in these events were interviewed to obtain data for demographic characteristics, clinical symptoms if SARS-CoV-2-positive and activity patterns.

## Laboratory techniques

The majority of SARS-CoV-2 RNA-positive samples were screened with an in-house variant-specific RT-PCR, targeting three mutations of interest: (i) L452R, (ii) E484K and (iii) E484Q. When none were detected, suggesting a variant which was not the Delta variant (Pango lineage B.1.617.2) predominant at the time in France, the whole genome was sequenced on an Oxford Nanopore Technologies MinION Mk1C device (ONT; Oxford, United Kingdom), using ARTIC LoCost V.3 protocol, or on a NextSeq500/550 Dx sequencing system, using Illumina COVIDSeq Test (Illumina, San Diego, United States (US)) [3]. The 20A clade/B.1.640 lineage was assigned after Nextclade and Pango lineage analysis [1,4]. All data were submitted to the EMERGEN Database [5].

## Identification and connection of clusters and events

**Figure 1:**
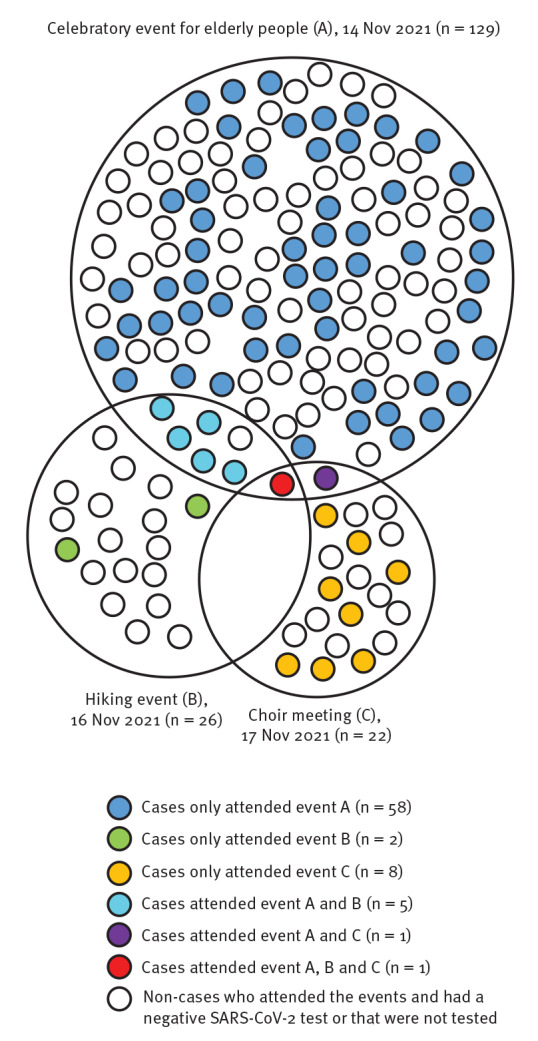
Links between three events related to confirmed and probable cases infected with SARS-CoV-2 B.1.640 variant, Normandy, France, November–December 2021

**Figure 2:**
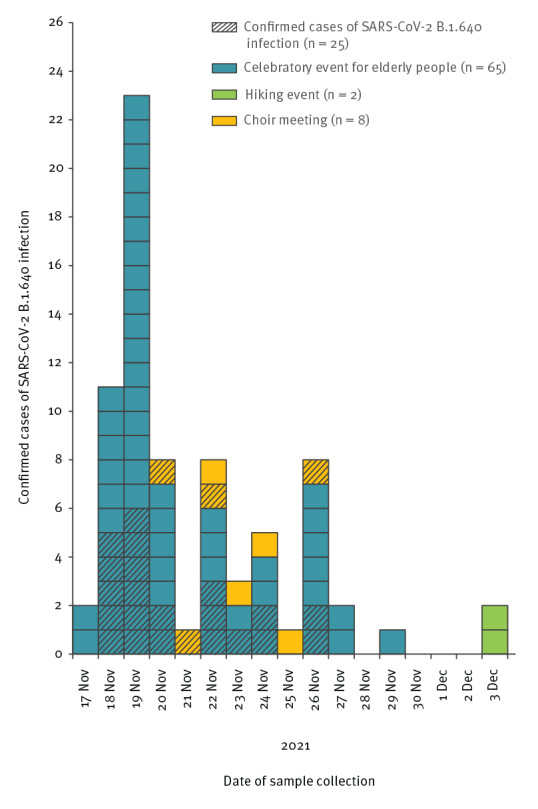
Distribution of confirmed and probable cases infected with SARS-CoV-2 B.1.640 variant, by date of sample collection, event affiliation and case classification, Normandy, France, November–December 2021 (n = 75)

The first event identified was a celebratory event for elderly people (event A). This event had 129 participants. All participants were required to have a complete primary vaccination schedule or past infection to participate. The event included a dinner followed by a dance party. The second event identified was an organised hike of 26 people (event B). Seven of the hikers had also attended event A 2 days before the hike. Carpooling was organised to get to the hiking site. Finally, a third event was identified, a choir meeting (event C) of 22 people who confirmed their presence, two of whom had also attended event A 3 days earlier. All of these events were predominantly attended by participants over 65 years of age. One case attended all three events (Figure 1). The time distribution of the confirmed and probable B.1.640 cases from each event is presented in Figure 2.

SARS-CoV-2: severe acute respiratory syndrome coronavirus 2.

SARS-CoV-2: severe acute respiratory syndrome coronavirus 2.

## Confirmed and probable SARS-CoV-2 B.1.640 cases and attack rates

Seventy-five individuals had a positive SARS-CoV-2 test. Among them, 52 (69%) had done an antigen test and 40 (53%) had done an RT-qPCR test. The tests screened with the variant-specific RT-PCR (n = 31) showed an absence of E484K, E484Q and L452R mutations, a profile compatible with the SARS-CoV-2 B.1.640 variant. Among those, whole genome sequencing confirmed the B.1.640 variant for 25 (33%) cases. These 75 confirmed and probable B.1.640 cases were distributed across three events, with event A accounting for 87% (n = 65) of these cases (Table). The overall attack rate (confirmed and probable B.1.640 cases) was 50.4% (65/129) and the secondary attack rate was 10.5% (2/19) for the hiking event and 40.0% (8/20) for the choir meeting.

**Table:** Demographic and clinical characteristics, according to case status in SARS-CoV-2 B.1.640 variant outbreak, Normandy, France, November–December 2021 (n = 168)

Categories	B.1.640 cases^a^ (n = 75)		Non-cases^b^ (n = 93)	
	n	%^c^	n	%^c^
Event				
Celebratory event for elderly people	65	86.7	64	68.8
Hiking event	2	2.7	17	18.3
Choir meeting	8	10.7	12	12.9
Total	75		93	
Test^d^				
Yes	75	100.0	57	80.3
No	NA		14	19.7
Antigen test	52	69.3	52	73.2
Rapid antigen self-test	0		2	2.8
RT-qPCR	40	53.3	3	4.2
Screening and sequencing				
Screening	31	41.3	NA	
E484K, E484Q and L452R mutations not detected	30	40.0	NA	
Cannot be interpreted	1	1.3	NA	
Whole genome sequencing	25	33.3	NA	
SARS-CoV-2 B.1.640 variant identified	25	33.3	NA	
Total	75		71	
Sex				
Female	45	60.0	57	61.3
Male	30	40.0	36	38.7
Total	75		93	
Age (years)				
Min	22.0		60.0	
Mean (SD)	72.4 (SD: 9.8)		73.0 (SD: 6.6)	
Max	92.0		91.0	
Total	75		70	
Symptoms				
Yes	56	82.4	6	8.1
No	12	17.6	68	91.9
Total	68		74	
Symptom onset before sample collection (days)				
0–1	11	30.6	NA	
2–4	21	58.3	NA	
5–7	3	8.3	NA	
>14	1	2.8	NA	
Total	36		NA	
Type of symptom^e^				
Runny nose	23	42.6	5	83.3
Asthenia	22	40.7	2	33.3
Cough	21	38.9	0	
Sore throat	15	27.8	1	16.7
Myalgia	14	25.9	1	16.7
Headache	12	22.2	0	
Fever	7	13.0	0	
Shortness of breath	4	7.4	0	
Ageusia	4	7.4	0	
Anosmia	3	5.6	0	
Diarrhoea	2	3.7	0	
Total	54		6	
Hospitalisation				
Yes	4	6.1	0	
No	62	93.9	74	100.0
Total	66		74	
Risk factors^f^				
Yes	29	50.0	33	44.6
No	29	50.0	41	55.4
High blood pressure	17	29.3	19	25.7
Diabetes mellitus type 1 or type 2	9	15.5	13	17.6
Heart conditions	6	10.3	5	6.8
Neuromuscular disease	1	1.7	0	
Respiratory disease	1	1.7	1	1.4
Liver disease	1	1.7	0	
Kidney disease	0		1	1.4
Cancer	0		1	1.4
Other	0		1	1.4
Total	58		74	
Vaccination history^g^				
Yes	65	100.0	70	100.0
No	0		0	
One dose^h^	2	3.1	3	4.3
Two doses	50	76.9	42	60.0
Booster dose	13	20.0	25	35.7
Total	65		70	
SARS-CoV-2 infection history				
Yes	2	3.1	10	13.9
No	62	96.9	62	86.1
Total	64		72	

NA: not applicable; SARS-CoV-2: severe acute respiratory syndrome coronavirus 2; SD: standard deviation.

^a^ Confirmed and probable cases of infection with the SARS-CoV-2 B.1.640 variant.

^b^ A non-case was any person with a negative SARS-CoV-2 test confirmed by RT-qPCR or antigen test or a person that was not tested.

^c^ Proportions are based on the number of responses provided.

^d^ Individuals may have done more than one test.

^e^ Individuals may have more than one symptom.

^f^ Individuals may have more than one risk factor.

^g^ Data on vaccination history could not be collected from all participants.

^h^ Equivalent to two doses because of previous SARS-CoV-2 infection.

## Demographic and clinical information

Sixty-two B.1.640 cases (confirmed and probable cases of infection with the SARS-CoV-2 B.1.640 variant) were over 65 years of age, with a mean age of 72.4 years (standard deviation (SD): 9.8) and 45 were women. The mean age was 73.0 years (SD: 6.6) for non-cases, of whom 57 were women. Among the 58 B.1.640 cases for whom information was available, 29 had at least one risk factor. The most common risk factors were high blood pressure (n = 17), diabetes mellitus type 1 or type 2 (n = 9) and heart conditions (n = 6). Of the 74 non-cases, 33 reported at least one risk factor, with the most common being high blood pressure (n = 19), diabetes mellitus type 1 or type 2 (n = 13) and heart conditions (n = 5). All B.1.640 cases and non-cases had a complete vaccination schedule or past infection, of whom 13 B.1.640 cases and 25 non-cases had already received one booster dose. The most frequently reported vaccine was Comirnaty (BNT162b2 mRNA, BioNTech-Pfizer, Mainz, Germany/New York, US) in cases (n = 47) and non-cases (n = 49). Two of 64 B.1.640 cases and 10 of 72 non-cases reported a previous SARS-CoV-2 infection (Table).

Fifty-six among the 68 B.1.640 cases with available information were symptomatic and 54 had documented symptoms. The most common symptoms were runny nose (n = 23), asthenia (n = 22), cough (n = 21), sore throat (n = 15), myalgia (n = 14) and headache (n = 12). Symptoms were experienced by six of 74 non-cases, most frequently runny nose (n = 5). Among the 66 B.1.640 cases with available information on hospitalisation, four were hospitalised, of whom two were B.1.640 confirmed cases. Three of the four hospitalised patients had risk factors.

Only one B.1.640 case had travelled abroad to a country outside Europe 2 weeks before attending event A. One B.1.640 case reported symptoms during the period before event A, but no ascertained at-risk contact was identified.

## Control measures

All confirmed and probable B.1.640 cases observed a 10-day quarantine at home as soon as they became aware of their own case status from the date of symptom onset or collection of the positive test. A screening operation took place on 27 November 2021 in the area where the three events occurred. The operation consisted of antigen testing of all people wishing to be tested and confirmation by RT-qPCR if antigen testing was positive.

### Ethical statement

The National Ethical Committee has granted the ARS and SpF approval to access personal data to investigate and control identified public health threats. No additional ethical clearance was required or sought.

## Discussion

The SARS-CoV-2 B.1.640 variant was first detected in late September 2021 in the Republic of Congo [1]. On 14 January, the GISAID database reported 450 submitted sequences, with more than 70% coming from France [6]. Although no studies are currently documented in the literature, SpF reclassified the SARS-CoV-2 variant B.1.640 as a variant of interest (VOI) in its risk assessment from early January 2022, based on in vitro investigations conducted by the Centre National de Référence Virus des infections respiratoires (National Reference Centre for Respiratory Viruses). These preliminary results suggested a reduction in the neutralisation of B.1.640 by post-infection or post-vaccination antibodies because of mutations and deletions observed in the S gene. These results should be interpreted with caution in view of the limited number of subjects and samples analysed. Additional analyses are in progress [7,8].

The overall attack rate and secondary attack rate for the choir meeting were probably exacerbated because of prolonged exposure, interactions in a confined indoor space and festive activities which may have favoured transmission. These factors have been widely described in findings on superspreading events [9,10]. A large number of the confirmed and probable B.1.640 cases were symptomatic, but most of them described mild symptoms and referred to their infection as a common cold. Only four people were hospitalised, three of whom had risk factors. One of these hospitalisations was probably not related to a SARS-CoV-2 infection. It is likely that vaccination had a role in reducing hospitalisation [11,12]. Although a third of the cases in our study group were confirmed as infected with the SARS-CoV-2 B.1.640 variant and none of the screened samples showed a profile not compatible with this variant, we cannot exclude the circulation of another variant.

The results of this investigation suggest substantial circulation of the B.1.640 variant, with symptomatic COVID-19 cases in a fully vaccinated population. However, these results should be interpreted with caution as the median age of those infected was over 65 years, therefore most participants were recently eligible to receive their booster dose. Studies suggest a substantial decrease of humoral response to the Comirnaty COVID-19 vaccine a few months after receiving the second dose, especially among people 65 years or older [13,14]. Furthermore, protection after the booster dose is not immediate and several confirmed and probable cases had received their booster dose less than 2 weeks before the positive test [15].

Between mid-November and mid-December, 153 sequences of SARS-CoV-2 B.1.640 variant, not all of which could be directly linked to these events were reported in Normandy. A probable chain of transmission was identified between a hospital cluster of confirmed cases with the SARS-CoV-2 B.1.640 variant and the celebratory event for elderly people (data not shown). It is likely that the investigated situation is the source of diffusion of this variant in the region.

## Conclusions

The results of this investigation indicate that the SARS-CoV-2 B.1.640 variant is transmissible among elderly people who have a complete primary vaccination schedule or one dose of vaccine and a past SARS-CoV-2 infection. However, given the small number of hospital admissions and considering the age of the confirmed and probable cases, vaccination may have had a role in reducing severity of illness. Given its continued circulation and reclassification to VOI in France, close surveillance and further studies are needed to better characterise clinical and epidemiological features of the SARS-CoV-2 B.1.640 variant and assess its impact on transmissibility, immunity and severity.

## References

[1] Cov-Lineages. Lineage B.1.640. PANGO lineages: latest epidemiological lineages of SARS-CoV-2. [Accessed: 13 Jan 2022]. Available from: https://cov-lineages.org/lineage.html?lineage=B.1.640

[2] World Health Organization (WHO). Tracking SARS-CoV-2 variants. Geneva: WHO. [Accessed: 13 Jan 2022]. Available from: https://www.who.int/en/activities/tracking-SARS-CoV-2-variants

[3] Protocols.io. nCoV-2019 sequencing protocol v3 (LoCost). Berkeley: protocils.io; 2020. Available from: https://www.protocols.io/view/ncov-2019-sequencing-protocol-v3-locost-bh42j8ye

[4] Nextclade. Clade assignment, mutation calling, and sequence quality checks. [Accessed: 20 Jan 2022]. Available from: https://clades.nextstrain.org

[5] Database EMERGEN. L'outil de dépôt du consortium EMERGEN. [The deposit tool of the EMERGEN consortium]. Saint-Maurice: Santé publique France. [Accessed: 20 Jan 2022]. French. Available from: https://emergen-db.france-bioinformatique.fr

[6] GISAID. Tracking of variants. [Accessed: 14 Jan 2022]. Available from: https://www.gisaid.org/hcov19-variants

[7] Sante Publique France. Analyse de risque sur les variants émergents du SARS-CoV-2 réalisée conjointement par Santé publique France et le CNR des virus des infections respiratoires. [Risk analysis on emerging variants of SARS-CoV-2 carried out jointly by Public Health France and the CNR for respiratory infection viruses]. 5 Jan 2022. Saint-Maurice: Sante publique France; 2022. French. Available from: https://www.santepubliquefrance.fr/media/files/01-maladies-et-traumatismes/maladies-et-infections-respiratoires/infection-a-coronavirus/analyse-de-risque-des-variants-emergents-de-sars-cov-2-05-01-22

[8] Sante publique France. Coronavirus: circulation des variants du SARS-CoV-2. [Coronavirus: circulation of SARS-CoV-2 variants]. 28 Jan 2022. Saint-Maurice: Sante publique France; 2022. French. Available from: https://www.santepubliquefrance.fr/dossiers/coronavirus-covid-19/coronavirus-circulation-des-variants-du-sars-cov-2

[9] Lee H Han C Jung J Lee S . Analysis of superspreading potential from transmission clusters of COVID-19 in South Korea. Int J Environ Res Public Health. 2021;18(24):12893. 10.3390/ijerph182412893 34948504 PMC8701974

[10] Brown CM Vostok J Johnson H Burns M Gharpure R Sami S Outbreak of SARS-CoV-2 infections, including COVID-19 vaccine breakthrough infections, associated with large public gatherings - Barnstable County, Massachusetts, July 2021. MMWR Morb Mortal Wkly Rep. 2021;70(31):1059-62. 10.15585/mmwr.mm7031e2 34351882 PMC8367314

[11] Botton J Dray-Spira R Baricault B Drouin J Bertrand M Jabagi M-J Reduced risk of severe COVID-19 in more than 1.4 million elderly people aged 75 years and older vaccinated with mRNA-based vaccines. Vaccine. 2022;40(3):414-7. 10.1016/j.vaccine.2021.12.009 34924220 PMC8664658

[12] Tenforde MW Self WH Adams K Gaglani M Ginde AA McNeal T Association between mRNA vaccination and COVID-19 hospitalization and disease severity. JAMA. 2021;326(20):2043-54. 10.1001/jama.2021.19499 34734975 PMC8569602

[13] Goldberg Y Mandel M Bar-On YM Bodenheimer O Freedman L Haas EJ Waning immunity after the BNT162b2 vaccine in Israel. N Engl J Med. 2021;385(24):e85. 10.1056/NEJMoa2114228 34706170 PMC8609604

[14] Levin EG Lustig Y Cohen C Fluss R Indenbaum V Amit S Waning immune humoral response to BNT162b2 Covid-19 vaccine over 6 months. N Engl J Med. 2021;385(24):e84. 10.1056/NEJMoa2114583 34614326 PMC8522797

[15] Patalon T Gazit S Pitzer VE Prunas O Warren JL Weinberger DM . Odds of testing positive for SARS-CoV-2 following receipt of 3 vs 2 doses of the BNT162b2 mRNA vaccine. JAMA Intern Med. 2022;182(2):179-84. 10.1001/jamainternmed.2021.7382 34846533 PMC8634151

